# Changes in the position of the medial meniscus owing to degenerative meniscus tears

**DOI:** 10.1016/j.asmart.2025.01.002

**Published:** 2025-01-22

**Authors:** Tomoyuki Kanayama, Yasushi Takata, Yoshihiro Ishida, Naoki Takemoto, Manase Nishimura, Satoru Demura, Junsuke Nakase

**Affiliations:** Department of Orthopedic Surgery, Graduate School of Medical Sciences, Kanazawa University, Kanazawa, Japan 13-1 Takara-machi, Kanazawa-city, 920-8641, Japan

**Keywords:** Degenerative tears, Medial meniscus extrusion, Meniscal tears, Osteoarthritis

## Abstract

**Background:**

While meniscal extrusion has been recognized as a key factor in meniscal dysfunction and osteoarthritis (OA) development, the specific movement of the posterior horn of the medial meniscus (MM) during extrusion, particularly in early-stage OA, remains unexplored. Therefore, in this study, we investigated the position of the MM in patients with medial knee pain and a Kellgren–Lawrence grade ≤1, investigating the relationship between meniscal extrusion and degenerative tears. We hypothesized that the MM extrusion (MME) would be larger when degenerative tears are present; the anterior horn would move posteriorly, and the posterior horn would move anteriorly, accordingly.

**Methods:**

A total of 181 knees (mean age 61.7 ± 12.1 years; 97 men and 84 women) were included. Simple radiographs were used to measure the weight-bearing line ratio and medial proximal tibia angle. Magnetic resonance imaging was used to measure the medial proximal tibia slope, medial meniscus extrusion, anterior and posterior horn position, and degenerative tears on the posterior segment of the medial meniscus. Those with degenerative tears were designated as group T and those without were designated as group C. Student's t-test and Pearson's χ^2^ test were performed to compare groups T and C. Statistical significance was set at p < 0.05.

**Results:**

Group T had a significantly larger medial posterior tibial slope (group T: 7.4 ± 2.3°; group C: 6.6 ± 2.2°, p = 0.010) and medial meniscus extrusion (group T: 2.7 ± 1.4 mm; group C: 1.9 ± 1.2 mm, p < 0.001) scores compared with group C. Furthermore, the posterior point of the anterior horn (group T: 16.3 ± 5.0 %; group C: 14.3 ± 3.8 %, p = 0.004) and anterior point of the posterior horn (group T: 36.4 ± 7.1 %; group C:26.9 ± 5.9 %, p < 0.001) were significantly larger in group T than in group C.

**Conclusion:**

Degenerative MM tears cause not only MME but also an anteroposterior shift.

## 1 Introduction

The meniscus has been the focus of several recent studies that investigated the initiation and progression of knee osteoarthritis (OA).^1^ Degenerative meniscal lesions develop slowly, primarily involve a horizontal tear of the meniscus, and are usually observed in middle-aged or older adult patients. Degenerative meniscal tears are a normal feature of aging and their prevalence increases with age.^2^

Injury to the meniscus can occur in many locations and is devastating, with many complications, including pain, instability, OA, and osteonecrosis.^3^ Recently, meniscal extrusion has been recognized as an important pathological state associated with meniscal dysfunction. Meniscal extrusion is the displacement of the meniscus medially or laterally, outside the margins of the tibial plateau. Some meniscal extrusion is physiological, with greater degrees thought to be pathological.^4^ Importantly, the pathologic degree of extrusion is associated with the development and progression of degenerative joint disease.^5^

**Fig. 1:**
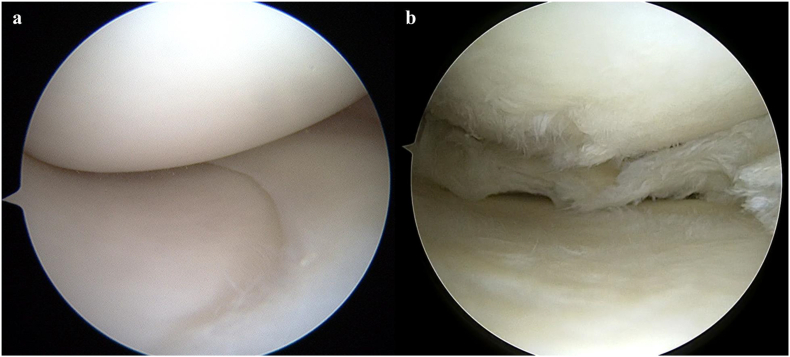
a, Arthroscopic finding without degenerative meniscus tear b, Arthroscopic finding with degenerative meniscus tear.

The medial meniscus (MM) is attached to the medial tibial plateau by the menisco-tibial ligament, forming the junctional structures of the meniscus and the tibial plateau, i.e., the anterior and posterior roots of the meniscus.^6^^,^^7^ The position of the anterior and posterior horn is expected to change when MM extrusion (MME) occurs. In knee OA, anterior meniscal extrusion associated with anterior tibial osteophytes is frequently observed in older populations.^8^ However, there have been no reports on the movement of the posterior horn. In cases of degenerative tears of the posterior horn of the MM, the position of the posterior horn is often depicted anteriorly on arthroscopy (Fig. 1). The requirement for surgery in patients with knee OA is determined based on age, activity, and alignment. Most meniscal lesions do not directly cause knee symptoms, and more than 60 % of tears are observed in individuals who do not experience knee pain, aches, or stiffness. However, symptomatic patients should be treated conservatively, and tears should be repaired.^9^ Therefore, in this study, we aimed to determine the MM position in patients with medial knee pain and a Kellgren–Lawrence (K–L) grade ≤1, which is not included in the general definition of OA. We hypothesized that the degree of MME would differ depending on the presence or absence of a degenerative tear in the MM. We also hypothesized that the MME would be larger in cases of degenerative tears, the anterior horn would move posteriorly, and the posterior horn would move anteriorly accordingly.

## 2 Materials and methods

This retrospective trial was approved by the ethics committee of our hospital (IRB No. 1842-3). All patients included in the study provided written informed consent prior to participation, in compliance with the principles of the Declaration of Helsinki. The study participants included consecutive patients who visited our hospital with the chief complaint of medial knee joint pain from April 2020 to March 2024, had a K–L grade ≤1, and had no history of ipsilateral lower extremity surgery. All patients underwent simple radiographic and magnetic resonance imaging (MRI) examinations. Finally, 181 knees (mean age 61.7 ± 12.1 years; 97 men and 84 women), excluding those with MM posterior root tears (MMPRT), were included in the study.

### 2.1 Radiographic evaluation

Radiographic OA severity was evaluated using the K–L classification based on the bilateral standing extended view of the weight-bearing anteroposterior radiographs of the tibiofemoral joint for both knees.^10^ The weight-bearing line ratio (WBLR) was determined using standing lower-extremity full-length radiography. To calculate the WBLR, a line was drawn from the center of the femoral head to the midpoint of the proximal talar joint surface. The WBLR was defined as the horizontal distance from the weight-bearing line to the medial edge of the tibial plateau, divided by the width of the tibial plateau.

### 2.2 MRI evaluation

**Fig. 2:**
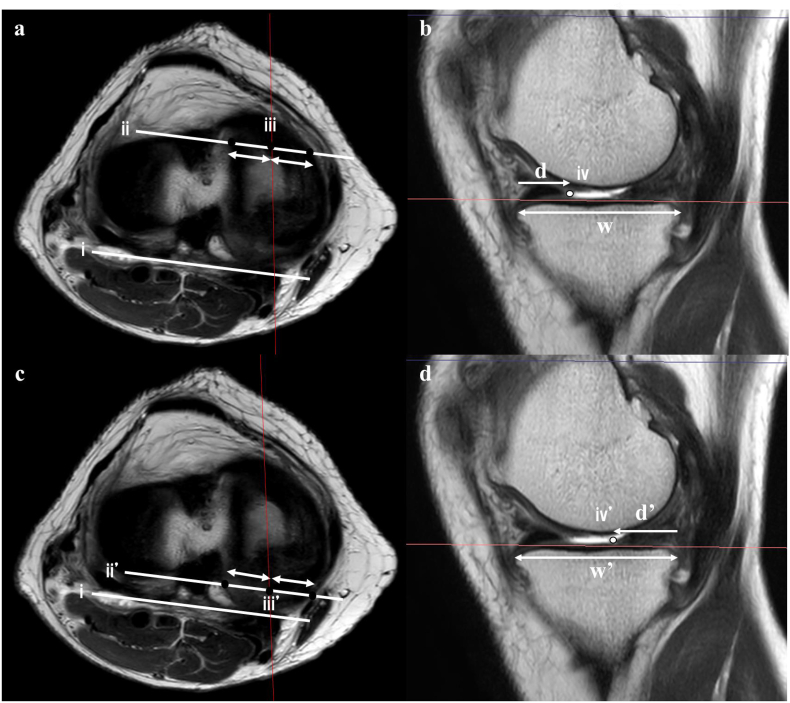
a, Measurement of the position of the anterior horn; coronal view i) a line of the posterior condyle ii) a parallel line of the posterior condyle through the anterior root attachment of the medial meniscus.iii) the midpoint of the tibial width on ii)b, Measurement of the position of the anterior horn; sagittal view through iii)iv) the distance from the posterior point of the medial meniscusw; the tibial width d, the distance from the posterior point of the medial meniscus c, Measurement of the position of the posterior horn; coronal viewi i’) a parallel line of the posterior condyle through the anterior root attachment of the medial meniscus.iii’) the midpoint of the tibial width on ii’)d, Measurement of the position of the posterior horn; sagittal view through iii’)iv’) the distance from the posterior point of the medial meniscusw’; the tibial width d’, the distance from the posterior point of the medial meniscus.

MRI was performed with a slight knee flexion. A 1.5-T MRI machine (Singa HDxt; GE, Boston, MA, USA) was used for image acquisition, with a 3-mm slice thickness and 1-mm interslice gap. The image data were analyzed using Synapse Vincent software (Fuji Films, Tokyo, Japan). Fat-saturated proton density-weighted MR was used to evaluate the MME in the coronal plane, where the medial collateral ligament is best depicted. The MME on MRI was defined as displacement from the margin of the tibial plateau and was measured as the distance between the margin of the tibial plateau and the peripheral border of the meniscal body. The medial posterior tibial slope (MPTS) was obtained from the sagittal MRI view following the method described by Hudek et al.^11^ First, a central sagittal image was chosen, and circles were drawn in the tibial head. The cranial circle touched the anterior, posterior, and cranial tibial cortical bones, and the caudal circle touched the borders of the anterior and posterior cortices. Subsequently, a line was drawn passing through the center of both circles, which was the longitudinal tibial axis. This line was copied to a sagittal slice where the medial tibial plateau could be identified, and the MPTS was calculated based on the angle between the line perpendicular to the longitudinal tibial axis and the line tangential to the subchondral bone of the medial tibial plateau. The position of the anterior horn was measured by first drawing a line along the posterior condyle (i), followed by drawing a parallel line passing through the anterior root attachment of the MM (ii). On this line, the midpoint of the tibial width (iii) was identified, and a sagittal image passing through this point was used for measurement (Fig. 2a). The tibial width (w) and the distance from the posterior point of the MM (iv) were measured in this slice (d), and the ratio was calculated as (d/w) × 100 (%) (Fig. 2b). Similarly, the position of the posterior horn was measured using a sagittal image passing through the midpoint of the tibial width (iii') on a parallel line (ii') passing through the posterior root of the MM (Fig. 2c). As in the previous section, the tibial width (w') and the distance (d') from the anterior point of the MM (iv') were measured in this slice, and the ratio was calculated as (d’/w’) × 100 (%) (Fig. 2d). Patients with Mink grade 3 horizontal tears in the posterior segment of the MM on MRI were classified into the degenerative tear group (Group T), whereas the others were classified as having no degenerative tears (Group C). All radiological parameters were measured twice by two observers (T.K. and Y.T.) with a 1-month interval between each measurement. The observers were blinded to the previous observations.

### 2.3 Sample size calculation and statistical analysis

The sample size was calculated using G-power 3.1 (with an effect size of 0.78, α error of 0.05, and target power of 0.95). Based on the MME data of a previous study,^12^ a minimum of 36 participants per group was recommended. All statistical analyses were performed using SPSS version 29.0 (IBM Corp., Armonk, New York, USA). The test-retest reliability was assessed using the intraclass correlation coefficient (ICC) and was analyzed using two-way random effects and absolute agreement. Student's t-test and Pearson's χ^2^ test were performed to compare age, sex, height, body weight, body mass index (BMI), K–L grade, WBLR, medial proximal tibial angle (MPTA), MPTS, MME, and the positions of the posterior point of the anterior horn and anterior point of the posterior horn between groups T and C. Statistical significance was set at p < 0.05. Post hoc tests were conducted using G-power 3.1 for which significant differences were observed.

## 3 Results

**Fig. 3:**
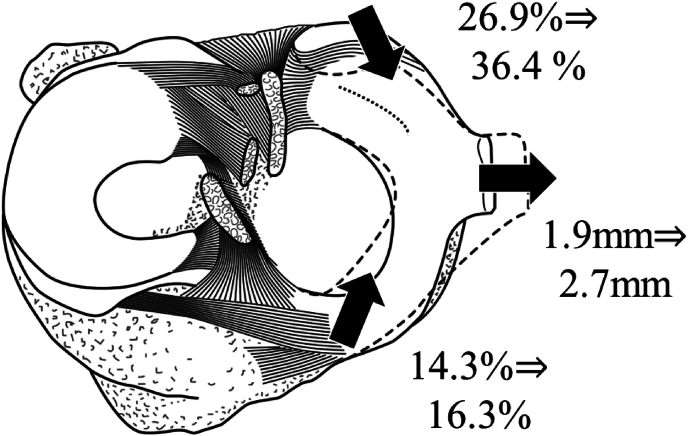
The schema for the position of the medial meniscus. Solid line: patients without degenerative tear (group C). Dotted line: patients with degenerative tear (group T).

The 181 patients were divided into two groups―105 patients in group T and 76 patients in group C. For the posterior point of the anterior horn, the ICC (1,2) was 0.927 (0.870–0.959) and the ICC (2,1) was 0.863 (0.770–0.920). For the anterior point of the posterior horn, the ICC (1,2) was 0.952 (0.915–0.973) and the ICC (2,1) was 0.908 (0.843–0.952). The results of the comparison between the two groups are presented in Table 1. The results showed no significant differences in age, sex, height, body weight, BMI, K–L grade, WBLR, or MPTA between the two groups. The schema for the position of MM is shown in Fig. 3. In group T, significantly larger MPTS (group T: 7.4 ± 2.3°; group C: 6.6 ± 2.2°, p = 0.010, power: 0.76) and MME (group T: 2.7 ± 1.4 mm; group C: 1.9 ± 1.2 mm, p < 0.001, power = 0.99) were observed, compared with group C. Furthermore, the posterior point anterior horn (group T: 16.3 ± 5.0 %; group C: 14.3 ± 3.8 %, p = 0.004, power = 0.91) and anterior point posterior horn (group T: 36.4 ± 7.1 %; group C:26.9 ± 5.9 %, p < 0.001, power = 1.00) were significantly larger in group T than in group C.

**Table 1:** Comparisons between the degenerative tear group and no degenerative tear group.

		Group T	Group C	P value
		n = 105	n = 76	
Age (years)		61.8 ± 11.7	61.6 ± 13.0	0.459
Male/female (knees)		55/50	42/34	0.309
Height (cm)		163.2 ± 8.	163.0 ± 8.4	0.437
Body weight (kg)		65.0 ± 13.2	62.9 ± 11.9	0.189
BMI (kg/m^2^)		24.3 ± 3.6	23.6 ± 3.4	0.134
K-L grade 0/1 (knees)		56/49	43/33	0.491
WBLR (%)		32.4 ± 12.1	21.1 ± 12.0	0.269
MPTA (°)		85.0 ± 2.2	85.2 ± 2.2	0.195
MPTS (°)		7.4 ± 2.3	6.6 ± 2.2	0.010
MME (mm)		2.7 ± 1.4	1.9 ± 1.2	<0.001
Posterior point anterior horn (%)		16.3 ± 5.0	14.3 ± 3.8	0.004
Anterior point posterior horn (%)		36.4 ± 7.1	26.9 ± 5.9	<0.001

Data are presented as mean±standard deviation.

Abbreviations: Group T, degenerative tear group; Group C, no degenerative tear group; BMI, body mass index; K-L, Kellgren-Lawrence; WBLR, weight-bearing line ratio; MPTA, medial proximal tibial angle; MPTS; medial proximal tibial slope; MME, medial meniscus extrusion.

## 4 Discussion

In this study, we assessed the position of the medial meniscus MM in patients with medial knee pain and a Kellgren–Lawrence grade ≤1. The most important finding of this study was that degenerative medial meniscal tears not only cause MME but also an anteroposterior shift. Future treatment strategies for early knee OA should consider not only the patient's MME but also changes in the anteroposterior shift.

Previous studies have reported the relationships between lower varus alignment, knee OA,^13^ and meniscal injury.^14^ Our results showed no significant differences in WBLR or MPTA with or without MM degenerative tears. Our study included patients with non-knee OA, suggesting that alignment abnormalities do not necessarily precede meniscal injury. Moreover, the MPTS was larger in group T than in group C. Heath et al.^15^ reported that increased PTS increased the MMPRT in cadaveric studies. An increased MPTS affected the compressive, tension, and medial shear forces of the posterior MM horn, which may have led to meniscal injury. Moon et al.^16^ reported a cutoff value of PTS of ≥6.6° as a risk factor for medial meniscus posterior horn injury. In the present study, the PTS was ≥6.6° in both groups, suggesting a contribution of the effect of tear, as well as the effect of PTS.

The results of this study revealed that the MME was greater in cases where a degenerative tear was present in the posterior horn of the MM than in cases without degenerative tears. Furthermore, Shimozaki et al.^12^ reported an MME of 2.5 ± 1.4 mm in patients aged approximately 61.6 years without MM injury. Lee CH et al.^17^ defined an MME <3 mm as a mild or physiological change and an MME ≥3 mm as abnormal. In group T, the MME was 2.7 mm, which was within the range of degenerative changes. Notably, the anteroposterior shifting of the meniscus, even within this range of degenerative changes, is interesting. When the position and morphology of the meniscus are altered, the mechanical forces on the articular cartilage are insufficiently distributed and the overall biomechanics of the knee joint are altered, resulting in cartilage loss and an increased risk of development and progression of knee OA.^18^ Therefore, it is important to restore the meniscus to its normal position during repair.

The anterior horn of the MM moves posteriorly, and the posterior horn moves anteriorly in patients with degenerative tears of the MM. Aditi et al.^8^ proposed a relationship between anterior meniscal extrusion and anterior tibial osteophyte formation. Under normal conditions, the MM attaches to the medial tibial condyle via the menisco-tibial ligament.^6^ However, in contrast to the tight linkage between the medial region of the meniscus and the tibial condyle by the ligament,^6^^,^^7^ the anterior region of the MM seems to be more loosely attached to the anterior tibial condyle by the anterior menisco-tibial ligament, and the medial part of the infrapatellar fat pad is located on the medial patellar ligaments.^6^^,^^19^ Therefore, when the width of the anterior tibial osteophyte is small, the edge of the anterior region of the MM may remain within the normal position of the meniscus without an anterior shift. However, in this study, the participants included patients with non-knee OA, and no significant differences in osteophytes were found. Meniscal tears are the strongest structural risk factor for the development of tibiofemoral osteophytes in middle-aged individuals with K–L grade 0.^20^

This study has some limitations. First, because this was a cross-sectional study, it is not clear whether meniscal shifts occurred before or after degenerative tears. Second, the location or size of the meniscal injury was not considered. Notably, meniscal shifts typically occur in the presence of severe tears, although they are expected to be more extruded in the presence of a larger meniscal injury. A previous study^21^ identified that medial meniscal root and radial tears were associated with an increased risk of concomitant MME with medial meniscus tear. Further research on the location and morphology of the tear is needed. Third, the definitive diagnosis of MMPRT was made using MRI rather than arthroscopy. Although this may not have completely ruled out MMPRT, the diagnostic power of MRI is adequate.^22^ Fourth, the MRI was taken with the knee in slight flexion, hence, because the MRI was taken in the unloaded state, this may have affected the displacement of the meniscus. However, previous reports^23^^,^^24^ found no difference in the amount of meniscus protrusion between unloaded and upright loading; therefore, we do not believe that the knee position during MRI would affect the results of this study. Finally, this was a two-dimensional measurement that did not examine the thickness of the meniscus. Despite these limitations, our finding regarding the location of the meniscus shifts/changes in patients with degenerative MM tears may influence future surgical strategies.

## 5 Conclusion

Degenerative MM tears cause not only MME but also an anteroposterior shift.

## Ethical approval

This non-randomized prospective controlled clinical trial was approved by the ethics committee of our hospital (IRB No. 1842-3).

## Informed consent

All patients provided informed consent prior to participating in the study.

## Funding sources

This research did not receive any specific grant from funding agencies in the public, commercial, or not-for-profit sectors.

## Declaration of interest statement

The authors did not receive support from any organization for the submitted work.

The authors have no conflicts of interest to declare that are relevant to the content of this article.

All authors certify that they have no affiliations with or involvement in any organization or entity with any financial interest or non-financial interest in the subject matter or materials discussed in this manuscript.
